# A pilot study on differential expression of microRNAs in the ventromedial prefrontal cortex and serum of sows in activity restricted crates or activity free pens

**DOI:** 10.5713/ajas.18.0910

**Published:** 2019-04-15

**Authors:** Guoan Yin, Liwei Guan, Langchao Yu, Dapeng Huang

**Affiliations:** 1 College of Animal Science and Veterinary Medicine, Heilongjiang Bayi Agricultural University, Daqing 163319, China

**Keywords:** Psychopathy, MiRNAs, Sow, Activity Restriction

## Abstract

**Objective:**

Physical activity restriction in sows may lead to behavioral abnormalities and affective disorders. However, the psychophysiological state of these sows is still unclear. As miRNAs can be used as effective markers of psychopathy, the present study aimed to assess the difference in microRNA expression between the long-term activity restricted sows and activity free sows, thus contributing to the understanding of abnormal sow behavior.

**Methods:**

Four dry sows (sixth parity, Large×White genetic line) were selected from activity restricted crates (RC) or activity free pens (FP) separately. microRNAs in the ventromedial prefrontal cortex (vMPFC) and serum were examined using real-time polymerase chain reaction, and the correlation between the miRNAs expressed in the vMPFC and serum was evaluated.

**Results:**

miR-134 (1.11 vs 0.84) and miR-1202 (1.09 vs 0.85) levels were higher in the vMPFC of the RC sows than in the FP sows (p<0.01). Furthermore, miR-132 (1.27 vs 1.08) and miR-335 (1.03 vs 0.84) levels were also higher in the RC sows than in FP sows (p<0.05); however, miR-135a, miR-135b, miR-16, and miR-124 levels were not different (p>0.05). The relative expression of serum miR-1202 was higher in the RC sows than in the FP sows (1.04 vs 0.54) (p<0.05), and there was a strong correlation (R = 0.757, p<0.05) between vMPFC and Serum levels of miR-1202. However, no significant difference was observed in miR-16 levels in the serum of the RC sows and FP sows (p>0.05).

**Conclusion:**

This pilot study demonstrates that long-term activity restriction in sows likely results in autism or other complex psychopathies with depression-like behaviors. These observations may provide new insights for future studies on abnormal behavior in sows and contribute to research on human psychopathy.

## INTRODUCTION

Psychopathy is a form of brain dysfunction that is affected by multiple genes and factors such as depression, schizophrenia, and autism. Psychopathy is mainly manifested in thought and behavioral disorders. It has been found that thought disorders can lead to positive or negative attention; this is especially true in patients with schizophrenia as there are positive symptoms such as indifference and escape and negative symptoms such as delusions and illusion [1]. However, the pathogenesis of most psychopathies is still unclear.

In recent years, the interest in researching microRNAs (miRNAs) has risen. Psychiatric research has also found that miRNAs are involved in approximately 70% of neuronal structural differentiation, neurotransmitter release, and synapse formation in the brain [2]. There are only 10 miRNAs regulate about 77% of presynaptic and 80% of post-synaptic protein transcription [3]. At least seven miRNAs are located on chromosome 8 [4], and slight changes in this region may induce psychopathy. However, miRNAs are differentially expressed in patients with psychopathy. For example, miR-135a is significantly downregulated in the brain and blood of patients with depression [5], and miR-132 is dysregulated in patients with Alzheimer’s and Parkinson’s disease [6]. Perkins et al [7] used a case-control study to analyze 264 miRNAs in the prefrontal cortex (PFC) of patients with schizophrenia and healthy controls. They found 16 miRNAs with differential expressions, 15 of which were significantly downregulated and one was upregulated. Moreover, depression can promote the upregulation of miRNA-let-7a expression in rats. Therefore, miRNAs can be used as an effective marker of psychopathy.

Previous studies of psychopathy-related miRNAs have focused on human and animal models; studies of animal psychopathy are few. As an emotionally rich animal, sows express affective disorders and behavioral abnormalities in adverse circumstances. Feeding level and physical restriction in sows may lead to stereotypic behaviors such as bar-biting and sham-chewing [8]; moreover, the pupillary light reflex (PLR) is significantly delayed [9]. The PLR is related to the stereotypes and neurotransmitters [10]. In terms of behavioral disorders, restricting sow activity can lead to the loss of social behavior and can also cause problems in the expression of maternal and defensive behaviors [11]. For example, sows with behavioral disorders response less to piglet screams and crush more piglets. Emotional instability is caused by abnormal behavioral defenses, and a higher sensitivity to stress is a combination of affective and attentional disorders, which is similar to bipolar disorder found in humans. Previous research has shown that activity restricted sows can present with mental disorders and may be in a depressed state [10]. Therefore, exploring the difference in expression of miRNAs will help to understand depression-like behaviors in sows. Moreover, miR-1202 [12], which was previously thought to exist only in primates was also detected in sows, indicating that sows have an emotional system similar to primates; therefore, the study on sows will also contribute to the progress of human psychopathy.

## MATERIALS AND METHODS

### Animal care

The experiment was reviewed and approved by the Animal Ethics Committee of College of Animal Science and Veterinary Medicine, Heilongjiang Bayi Agricultural University.

### Animals and treatment

The experiment was conducted on the Swine Farm of Heilongjiang Animal Science Institute in Qiqihar, Heilongjiang Province. Four dry sows (sixth parity, with Large×White genetic line) were selected from activity restricted crates (RC) or activity free pens (FP) separately. The two samples of sows were of similar weight and health.

The RC sows were housed in crates (2,100 mm×600 mm) throughout the entire gestation period and in crates with a 60 cm width sow stall (2,150 mm×600 mm) during lactation. The crates were made of stainless steel, and there were doors on the front and rear. The FP sows were housed together with a pen-mate in pens (3,000 mm×2,000 mm) during gestation, and farrowing in a straw pen (2,000 mm×2,000 mm) with a creep area. Natural ventilation and lighting were adopted in the house. The house was cleaned daily to ensure that it was dry and clean, sows were fed at 6:00 and 16:00.

### Sample collection

The blood sample were collected at 5:30, the sows were fixed, and seventy-five percent alcohol was used to find the most obvious blood vessels behind the ear; using a 6th blood collection needle and vacuum condensation tube, ear vein blood was collected from the sows with minimal stress. The collected blood stood at room temperature for 2 h, and was then centrifuged (2,000 rpm, 10 min) in a low-temperature, high-speed centrifuge. The serum was dispensed into a RNase-free EP tube via a pipette and stored in a refrigerator at −20°C.

The brain samples were collected at 22:00. After the sows were euthanized by electrical stunning, the brain was rapidly extracted and hippocampal tissue and the ventral prefrontal lobe were dispensed into a RNase-free cryotube with a sterilized scalpel, and two replicates were taken for each tissue. The prepared samples were stored in liquid nitrogen and transported back to the Northeast Agricultural University laboratory stored at −80°C for subsequent miRNA extraction.

### RNA extraction

Total RNA was extracted from the ventromedial PFC (vMPFC) region and blood was extracted with TRIzol reagent (Life Technologies, Carlsbad, CA, USA). RNA concentration and purity was determined using an ultra-micro spectrophotometer K5600, and detection of RNA integrity was with agarose gel electrophoresis.

### cDNA synthesis

The reaction system was prepared according to instructions by ReverTra Ace qPCR RT Kit (Sangon Biotech, Shanghai, China), and then reverse transcription reaction was carried out according to the following reaction conditions: 37°C, 15 min → 98°C, 5 min → 4°C, ∞. After the reaction was completed, the obtained cDNA solution was stored at −20°C.

### Quantitative real-time polymerase chain reaction (qPCR)

The internal reference for the primers (Scientia Biotech, Harbin, China) was U6 (U6 downstream primer as a general downstream primer), the primer sequences are shown in (Table 1). The cDNA concentration was measured and quantified to 100 ng/μL. According to the instruction of SYBR qPCR Mixv (Sangon Biotech, China), the quantitative real-time polymerase chain reaction (qPCR) reaction system was prepared, and samples were centrifuged and gently bombed twice. The program was set up on the PCR machine (BioRad, Hercules, CA, USA), and data were collected and exported.

### Statistical analysis

Using SPSS Statistics version 20 for data analysis, a normal distribution test was performed. The data conform to the normal distribution, and analyzed with a one-way analysis of variance. Correlation analysis between miRNAs content in the vMPFC and serum was conducted using Pearson’s correlation. The statistical results are presented as mean±standard deviation.

## RESULTS

### Total RNA test results

The results of the RNA electrophoresis showed that the brightness of 28 S and 18 S was larger than 5 S, and the analysis results of the ultra-micro spectrophotometer K5600 showed that the optical density 260/280 values were all in the range of 1.8 to 2.1.The extracted RNA had no obvious contamination and degradation, which can be used for cDNA.

### Expression of miRNAs in the vMPFC

**Figure 1:**
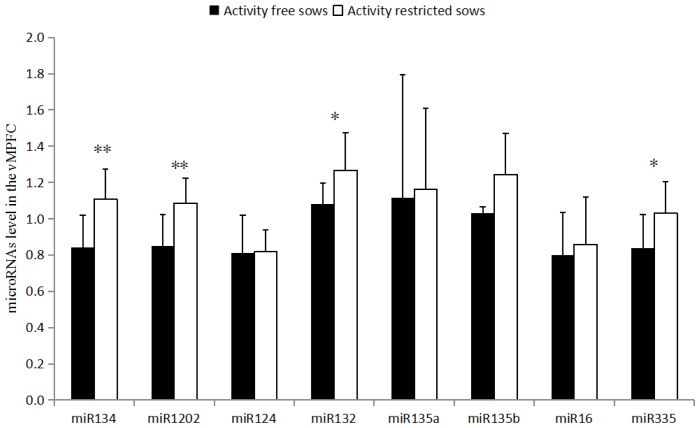
Levels of miRNA in the ventromedial prefrontal cortex. The data are presented as the means±stand error of mean, and n = 4 for each group. *, ** Means with significant differ (p<0.05) and (p<0.01).

As shown in (Figure 1), miR-134 and miR-1202 levels in the vMPFC of the RC sows are higher than that of the FP sows (p<0.01). miR-132 and miR-335 levels are also higher in RC sows than in FP sows (p<0.05). miR-135a, miR-135b, miR-16, and miR-124 levels were not different (p>0.05).

### Expression of miRNAs in the serum

**Figure 2:**
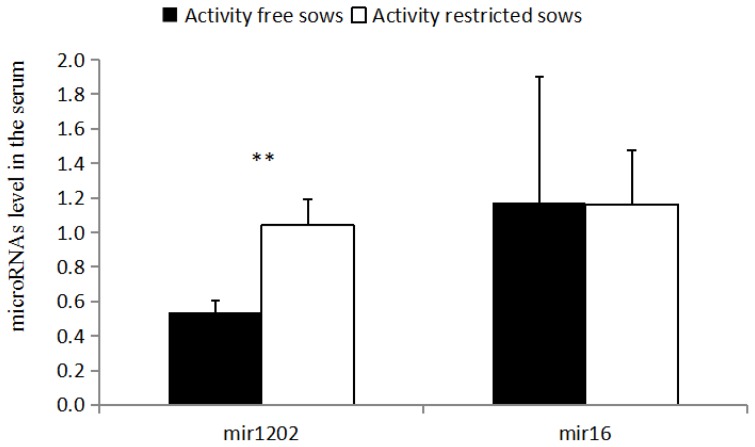
Levels of miRNA in the serum. The data are presented as the means±stand error of mean, and n = 4 for each group. ** Means with significant differ (p<0.05) and (p<0.01).

As shown in (Figure 2), the relative expression of serum miR-1202 in RC sows is higher than that of the FP sows (p<0.05), and there is no significant difference between miR-16 levels in the serum of the RC sows and FP sows (p>0.05).

### Correlation of miRNAs in the vMPFC and serum

There is a strong correlation (R = 0.757, p<0.05) between vMPFC and Serum levels of miR-1202, while there is no significant correlation (p>0.05) for miR-16.

## DISCUSSION

The medial PFC regulates the neural circuits of the hypothalamic-pituitary-adrenal axis and emotional response, and stress can lead to a decrease in the number of astrocytes in the vMPFC and reduction of dendritic spines length [13]. In this study, we demonstrated that activity restriction can reduce stress tolerance and lead to upregulation of miRNAs in the serum and vMPFC of sows.

miR-335 is involved in the development of a variety of neu rological diseases, and is commonly downregulated in patients with depression [14]. Moreover, miR-335 can directly target glutamate metabotropic receptor 4 (GRM4) to regulate cyclic adenosine monophosphate (cAMP) [14]. GRM4 is related to the regulation of anxiety-related behaviors [15] and can further regulate the expression of miR-335 [14]. The results of this study showed that the level of miR-335 in the vMPFC of RC sows was higher than that of the FP sows (p<0.05), while there was downregulation of this gene in depressed patients [14]. However, this finding contradicts the hypothesis that sows in the activity restricted environment are in a state of depression. Several studies [16] have described the upregulation of miR-335 in patients with schizophrenia and autism, implying that restricting movement may lead to schizophrenia or autism.

miR-134 is a brain-specific miRNA that negatively regulates dendritic spine development and maturation by encoding the LIM domain kinase 1 (LIMK1) [17]. In this study, the detection of miR-134 in the vMPFC of RC sows was higher than that of the FP sows (p<0.01). Overexpression of miR-134 inhibited the translation of LIMK1 mRNA, shortened the dendritic spine, and reduced the sensitivity of the animal to stress [17], which may be related to the PLR in the activity restricted environment [18]. Such pathways can modulate chronic stress-induced structural plasticity and depression-like behaviors [19]. Patients with depression [19] and autism demonstrate upregulated expression of miR-134, whereas patients with bipolar disorder and *in vivo* miRNA in patients with schizophrenia [20] reveal a downregulation of miR-134 expression in the blood (p<0.05) [21]. Thus, the results of miR-134 support the hypothesis that RC sows may have depression or autism, while negating the speculation of schizophrenia.

miR-132 is closely related to the occurrence of neurological diseases, and also plays an important role in the connection between the nervous and immune systems. Moreover, miR-132 can inhibit the translation of RAS p21 protein activator 1. mRNA positively regulates axonal elongation [22], and miR-132 activates the hippocampal brain derived neurotropic factor (BDNF)-extracellular signal-related kinases-cAMP response element-binding protein signaling pathway by promoting the upregulation of BDNF [23], thereby promoting neuronal growth. The results of the present study showed that the level of miR-132 in the vMPFC of RC sows was higher than that of the FP sows (p<0.05). The same result is also observed in fetal malformed brain tissue and brains of patients with epilepsy [24]. miR-132 can promote neuronal growth and development in a variety of ways, but abnormal upregulation of miR-132 can induce epilepsy, mild cognitive impairment, and other neurological diseases. Studies have shown that patients with autism and depression display upregulated miR-132 expression in vivo, while miR-132 is downregulated in patients with schizophrenia [25]. The upregulated expression of miR-132 in the vMPFC suggests that the RC sows may exhibit symptoms of depression or autism.

miR-1202 is a newly discovered depression-related transcriptional regulator, and we demonstrated that there was strong correlation of mir-1202 in the vMPFC and serum, the similar research was found in depression patients[26]. miR-1202 is downregulated in patients with depression, and is associated with the GRM4 3′UTR region [26]. Binding regulates the metabolism of glutamate, miR-1202 participates in the regulation of the structure and function of the animal nervous system. Previous research suggests that it only exists in primates [27], and miR-1202 has been reported in other studies of psychopathy. The level of miR-1202 in the vMPFC and serum of the RC sows was higher than in the FP sows (p<0.01), which may be due to the downregulation of GRM4 [27]. Studies have found that miR-1202 and GRM4 may be involved in the regulation of glutamate neurotransmitter metabolism [28], and thus affecting neurotransmitter release and the mediation of neural signaling pathway transduction. The level of peripheral miR-1202 is associated with changes in brain activity, which affect a wide range of neural networks, including the prefrontal, temporal, and parietal cortices, which can affect mood and cognition [28]. Therefore, the upregulation mechanism of miR-1202 may lead to the irritability of sows, one of the main reasons for vulnerability to stress.

miR-124 is important in the differentiation and regener ation of neurons, which inhibits the expression of BDNF in the hip joint cavity of depressed rats. The results of the present study showed that there was no significant difference in the expression levels of miR-124, which is inconsistent with the upregulation of miR-124 expression in hippocampus of previously reported depressed rats [29]. miR-135a and miR-135b regulate serotonergic activity, and serotonin has therapeutic effects in depression and anxiety. Previous studies have found that when mice were administered antidepressants, miR-135a was significantly upregulated, while mice with dysregulated miR-135 expression exhibited anxiety and depression-like behaviors [30]. However, the present study did not find any significant differences between miR-135a and miR-135b in the two groups of sows. Moreover, miR-16 can be involved in the regulation of serotonergic activity as a negative regulator, and miR-16 has been found to be upregulated in patients with schizophrenia. There was no significant difference in the expression of miR-16 between the two groups in serum and vMPFC.

## CONCLUSION

The downregulation of microRNAs in the vMPFC and serum suggests that long-term activity restriction may affect the structure of the vMPFC, which may lead to an increase in abnormal behaviors. Furthermore, serum miRNA levels can be used for studies on sow psychophysiology. This pilot study supports the hypothesis that restriction of long-term activity in sows might result in autism or other complex psychopathy with depression-like behaviors. However, further extensive research is still required to confirm our observations.

## Figures and Tables

**Table 1:** Primer sequences

Primer name	Sequence
MIR16-F	ATAGCAGCACGTAAATATTGGCG
MIR135a-F	CGCGTATGGCTTTTTATTCCTATGTGA
MIR1202-F	GTGCCAGCTGCAGTGG
MIR124-F	TAAGGCACGCGGTGAATG
MIR132-F	TAACAGTCTACAGCCATGGTCG
MIR134-F	TGTGACTGGTTGACCAGAGG
MIR335-F	CGCGTCAAGAGCAATAACGAAAAATG
MIR135b-F	CCGTATGGCTTTTCATTCCTATGTGA
U6-F	CTCGCTTCGGCAGCACA
U6-R	AACGCTTCACGAATTTGCGT
